# P- and R-wave Amplitude Sensed by Reveal LINQ™ Loop Recorder in Pediatric Patients

**DOI:** 10.19102/icrm.2017.080102

**Published:** 2017-01-15

**Authors:** Roshan D’Souza, Elizabeth Thomas, Scott Macicek, Peter Aziz, Jill K. Shivapour, Christopher Snyder

**Affiliations:** ^1^Rainbow Babies and Children’s Hospital, Case Western Reserve University School of Medicine, Cleveland, OH; ^2^Oschner Medical Center, New Orleans, LA; ^3^Cleveland Clinic Children’s Hospital, Cleveland, OH

**Keywords:** Arrhythmia, congenital heart disease, loop recorder, R-wave amplitude

## Abstract

Implantable loop recorders are commonly used to sense arrhythmias. The purpose of this study is to assess the P- and R-wave amplitudes at implantation (I) and follow-up (F) following insertion of the Reveal LINQ™ Insertable Cardiac Monitor (Medtronic, Minneapolis, MN) in an institutional review board-approved, multicenter study performed on pediatric patients younger than 18 years old. Collected data included demographics, presence of congenital heart disease (CHD), P- and R-wave-sensed amplitude at I and F, and the method of implant (i.e. mapping or standard.) P waves were manually measured and R-wave sensing was recorded by the device. A total of 87 patients had a Reveal LINQ™ (Medtronic, Minneapolis, MN) device implanted; the mean patient age was 11.8 years (0.5 years to 18 years) with 48% of patients being female and 19% of patients having CHD; mapping was used in 43% of patients. The Reveal LINQ™ (Medtronic, Minneapolis, MN) experienced no change in average sensed R-wave amplitude at either I or F (1.28 mV vs 1.26 mV, p = NS). There was no difference in sensed R-wave amplitude noted with or without mapping used at I (1.29 mV vs 1.26 mV, p = NS) or F (1.48 mV vs 1.18 mV, p = NS). Additionally, no difference could be found in R-wave sensing of patients with CHD or without CHD at I (1.26 mV vs 1.4 mV, p = NS) or F (1.32 mV vs 1.32 mV, p = NS). R-wave sensing trended towards being inversely proportional to patient body surface area (BSA) (p = NS). P waves were detected on 48% of tracings in all patients at I and/or F, irrespective of whether the Reveal LINQ™ (Medtronic, Minneapolis, MN) device was placed with mapping. The R wave was (0.37–3.5 mV) at I and (0.3–3 mV) (p = NS) at F when P waves were detected. From these results, it can be said that the Reveal LINQ™ Insertable Cardiac Monitor (Medtronic, Minneapolis, MN) has an excellent ability to sense R-wave amplitude in pediatric patients. No significant difference in the sensing ability of the device could be identified with respect to the presence of CHD, use of mapping or BSA. P waves tended to be identified when there was a higher baseline R-wave amplitude.

## Introduction

An implantable loop recorder (ILR) is an ambulatory device used for the detection of arrhythmias over a prolonged period of time.^1^ A novel ILR, the Reveal LINQ™ Insertable Cardiac Monitor (Medtronic, Minneapolis, MN), has been approved by the United States Food and Drug Administration for use in patients with chest pain, syncope, and/or dizziness.^2,3^ The advantages of this new ILR are its small size and streamlined delivery system, which allows for implantation outside of the cardiac catheterization laboratory.^4–6^

The Reveal LINQ™ (Medtronic, Minneapolis, MN) also has an improved algorithm for the detection of both P waves and R waves, which correspond to the signals for atrial and ventricular depolarization, respectively, on the standard electrocardiogram.^7,8^ This algorithm has been validated in adult patients to assist in P-wave detection.^9,10^ Further research has demonstrated that a sensed R-wave amplitude of at least 0.2 mV is required for arrhythmia detection.^11^

The first case report of a Reveal LINQ™ (Medtronic, Minneapolis, MN) device being implanted into a pediatric patient described successful use in an 11-month-old infant.^12^ However, there are no published data on the use of the device in conjunction with arrhythmia detection or sensing of P or R waves in pediatric patients. Therefore, the purpose of this study is to assess the P and R wave amplitudes sensed at implant and follow-up of the Reveal LINQ™ (Medtronic, Minneapolis, MN) device in pediatric patients.

## Methods

A multicenter institutional review board approved the study of data from patients younger than 18 years of age; the data were retrospectively evaluated from February 2014 to January 2016. The data included demographics, the presence of congenital heart disease (CHD), patient body surface area (BSA), and the location of implant, either after pre-implant mapping or the standard fourth intercostal space. P- and R (QRS)-wave-sensed amplitude at implantation (I) and follow-up (F) was greater than 1 month. P waves were manually measured and R-wave sensing was recorded by the device.

**Figure 1:**
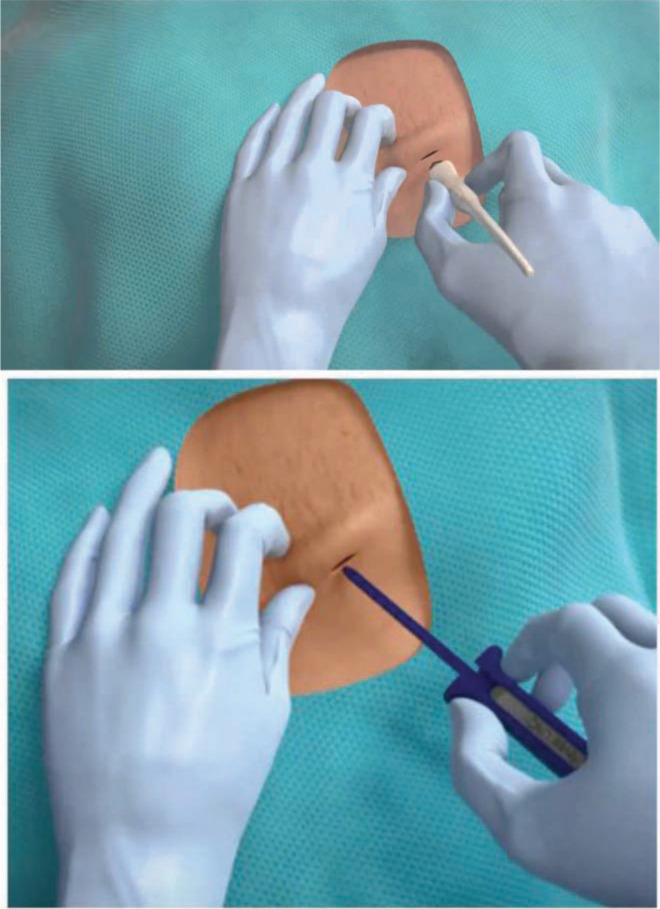
The first image demonstrates a stab incision made with the incision tool and followed by the Loop Reveal LINQ™ Recorder (Medtronic, Minneapolis, MN) being placed through the incision using the delivery system in the subcutaneous pocket.

The standard implant site is the left fourth intercostal space. The Reveal LINQ™ (Medtronic, Minneapolis, MN) device comes with a preloaded tool. A stab incision less than 1 cm is made with the incision tool in the fourth intercostal space 2 cm away from the patient’s sternum at a 45-degree angle after bupivacaine infiltration **(Figure 1)**. The Reveal LINQ™ (Medtronic, Minneapolis, MN) device is placed through this incision using its delivery system into the subcutaneous pocket. The small incision is then closed with Monocryl^®^ subcuticular sutures (Ethicon, Somerville, NJ) and a light dressing is subsequently placed on the left chest area. Individual variations that include change in location or in angulation of the device were either due to low voltage in the pre-implant surface mapping or as dictated by the patient’s cardiac anatomy.

Surface mapping was conducted at selected institutions with clinic electrocardiogram equipment or with a CareLink^®^ 2090 Programmer (Medtronic, Minneapolis, MN). The surface mapping was believed to improve the R-wave detection. The R-wave amplitude was either detected by the programmer or manually measured on the printouts. The detection of P waves was performed via visualization of the device printout at I and F by a single blinded observer. Any complications were recorded for assessment of the safety profile.

The results were analyzed using the SAS version 9.4 (SAS Inc., Cary, NC) package for average mean, standard deviation, Pearson correlation coefficient, Wilcoxon two-sample test, paired t-tests, and unpaired t-tests.

## Results

A total of 87 patients underwent Reveal LINQ™ (Medtronic, Minneapolis, MN) device implantation, with 59 of them having both I and F data. The mean age at the time of I was 11.8 years (0.5 years to 18 years) **(Table 1)**. Forty-eight percent of these individuals were female and 19% of the total patient group had CHD. Indications for implant included syncope (48%), palpitations (25%) and other (27%). All patients tolerated the procedure without complications.

**Figure 2:**
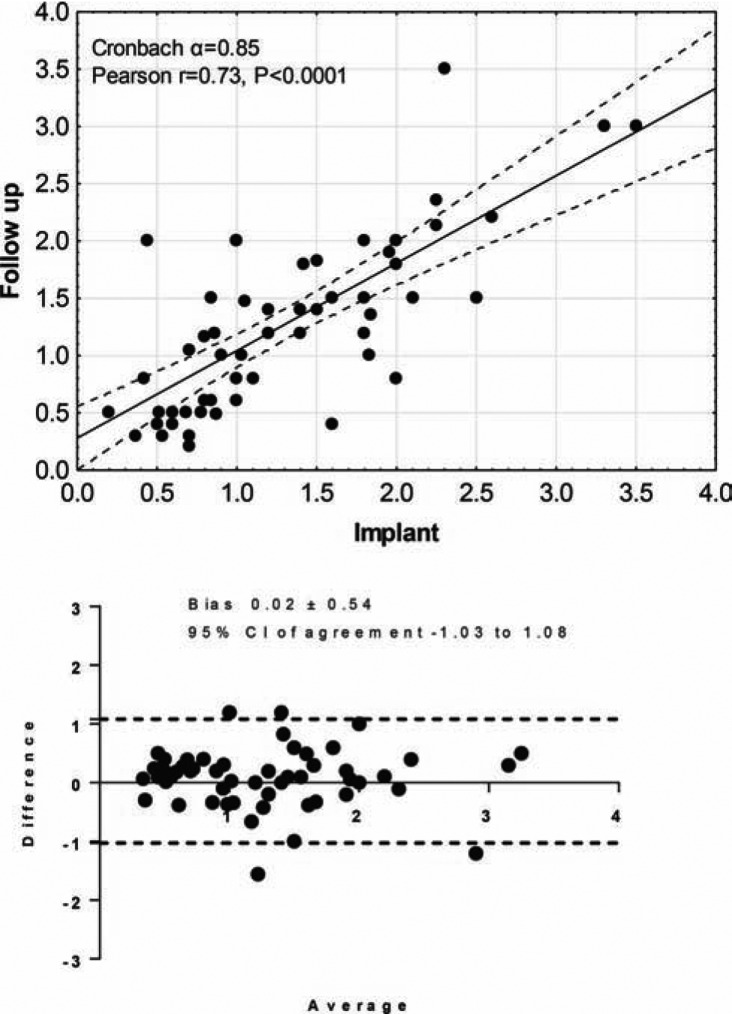
The first graph demonstrates the correlation of R-wave amplitude at implant and follow-up. Both R wave at implant and at follow-up were inversely associated with age. Spearman r = -0.29, p = 0.02, and at follow-up r = -0.29, p = -0.02. The next graph shows 95% confidence interval agreement of R wave at implant and at follow-up.

The average sensed R-wave amplitude recorded by the Reveal LINQ™ (Medtronic, Minneapolis, MN) device at I was 1.28 mV (0.2–3.5 mV), with no decrease noted at F 1.26 mV (0.2–3.5 mV) (p = NS). Correlation of R-wave amplitude at I and F showed 95% confidence interval agreement of R wave at I and F **(Figure 2)**. Pre-implant mapping for the ideal sensing location on the chest was used in 43% of the procedures; no difference was noted in the sensed R-wave amplitude between those using the standard implant technique at I (1.29 mV vs 1.26 mV) or F (1.48 mV vs 1.18 mV) (p = NS). In addition, there was no difference detected in sensed R wave in patients with or without CHD at I (1.26 mV vs 1.32 mV) or F (1.4 mV vs 1.32 mV).

Following implantation, the Reveal LINQ™ (Medtronic, Minneapolis, MN) tracings were analyzed to identify P waves. We detected P waves on the printouts of 39 of the 87 (45%) patients. The average R-wave amplitude at I when P waves were detectable was 0.37–3.5 mV, compared with 0.3–3 mV when no P waves and no difference was noted at F (0.2–3.3 mV vs 0.2–3 mV, p = NS). Furthermore, P waves were detected in 38% (n = 14) of the patients who underwent pre-implant mapping compared to 50% (n = 25) in which no mapping was used (p = NS).

**Figure 3:**
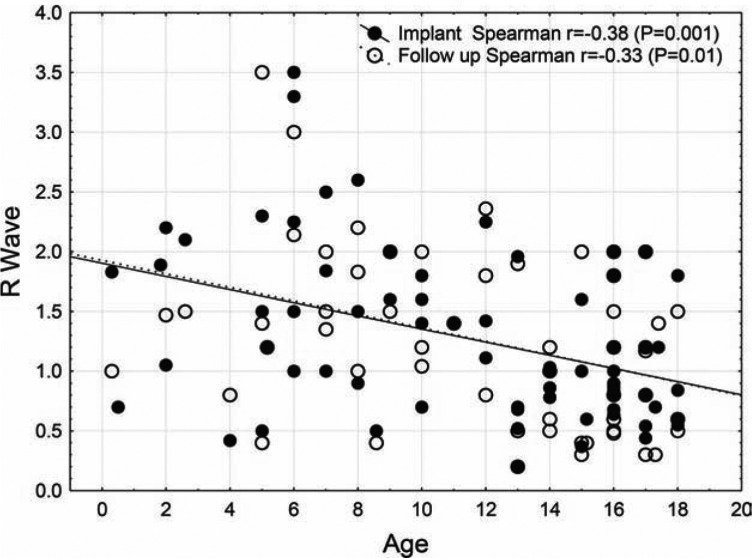
Association between age and R waves at implant and at follow-up. Both R wave at implant and at follow-up were inversely associated with age. Spearman r = -0.29, p = 0.02, and at follow-up r = -0.29, p = 0.02.

Comparisons of sensed R-wave amplitude based on the patient BSA found that those with BSA greater than 2 m^2^ had a lower average R-wave amplitude (0.82 mV) at I when compared to those with BSA of 0.5–1 m^2^ at (1.63 mV). The same difference in R wave sensing was present at F (0.66 mV vs 1.77 mV), with neither the BSA >2 m^2^ nor the BSA of 0.5–1 m^2^ reaching statistical significance. In patients with a BSA of less than 0.5 m^2^ (n = 2), the average R-wave amplitude at I measured 1.13 mVand was 1.23 mV at F, yielding excellent results in these smaller patients of under 1 year of age **(Figure 3)**. No complaints occurred in these smaller patients.

## Discussion

The Reveal LINQ™ (Medtronic, Minneapolis, MN) ILR algorithm for the detection of atrial and ventricular arrhythmias is based on the accurate sensing of P and R waves. This study demonstrates that the sensed R-wave amplitude in pediatric patients—regardless of BSA, method of implantation, and/or the presence of CHD—was acceptable at both I and F, based on the minimum R-wave sensing threshold of 0.2 mV published by Medtronic. In patients with a BSA of less than 0.5 m^2^, the average amplitude at I and F was similar to the rest of the group. In an adult study, 30 patients were evaluated with the R-wave amplitudes at implantation of 0.58 ± 0.32 mV and 0.59 ± 0.33 mV at 1 month (p = NS).^11^ Our pediatric study cohorts demonstrated a higher R-wave amplitude at I and F.

P waves were detected by the Reveal LINQ™ (Medtronic, Minneapolis, MN) device in nearly half of the patient cohort. Again, our sub-analysis on pre-implant mapping did not statistically improve the visualization of P waves compared with the standard implant. A similar conclusion was derived in adult cohorts, suggesting that pre-implant mapping may not be necessary.^13^ When the P waves were detected, the R-wave amplitude trended higher than the patients with no P waves.

The pediatric implantation technique is similar to that of the adult method; however, the procedure is done under local anesthesia in adults. Pediatric patients, in contrast, require the use of deep sedation with or without intubation and/or the use of advanced airway, necessitating the procedure to be performed in the catheterization lab or procedure room.

### Study limitations

The retrospective nature of data collection limits this study. Only 59 patients out of the total 87 had complete data at implant and follow-up, and hence were included in the data analysis so as to avoid potential selection bias.

## Conclusions

The Reveal LINQ™ (Medtronic, Minneapolis, MN) device yielded good R-wave (QRS) amplitude in pediatric patients. No significant difference was noted based on the presence of CHD, pre-implant mapping, or BSA. P waves were visualized in patients with higher R-wave amplitude with no significant difference observed because of pre-implant mapping. The device can be implanted safely in smaller patients.

## Figures and Tables

**Table 1:** Age and BSA Data

Variable	Total	Mean	SD	P
Age	87	12.3 years	5.2	
BSA	87	1.4 m^2^	0.48	
**R wave amplitude**				
R waves in all patients at implant	59	1.28 mV	0.72	
R waves in all patients at follow up	59	1.26 mV	0.75	0.72
R waves in patients with CHD at implant	8	1.26 mV	0.78	0.29
R waves in patients with pre-implant mapping at implant	30	1.29 mV	0.72	0.59
R waves in patients with pre-implant mapping at follow-up	39	1.26 mV	0.66	0.74
R waves in patients with pre-implant mapping when P waves were detected at implant	33	1.19 mV	0.64	0.26
R waves in patients with pre-implant mapping when P waves were detected at follow-up	32	1.3 mV	0.74	0.8

BSA: body surface area; CHD: congenital heart disease; F: follow-up; I: implant.The p-value (p0.05) was not significant when the R-wave amplitude data were compared between all patients at I and F, with or without CHD at I, with or without mapping at l/F, and when P waves were detected at l/F with or without mapping.
